# Psychophysiology of positive and negative emotions, dataset of 1157 cases and 8 biosignals

**DOI:** 10.1038/s41597-021-01117-0

**Published:** 2022-01-20

**Authors:** Maciej Behnke, Mikołaj Buchwald, Adam Bykowski, Szymon Kupiński, Lukasz D. Kaczmarek

**Affiliations:** 1 Adam Mickiewicz University, Faculty of Psychology and Cognitive Science, Poznan, 61-664 Poland; 2 Wrocław University of Science and Technology, Faculty of Information and Communication Technology, Wrocław, 50-370 Poland; 3 Poznan Supercomputing and Networking Center, Network Services Division, Poznan, 61-139 Poland

**Keywords:** Psychology, Psychology, Cardiovascular biology

## Abstract

Subjective experience and physiological activity are fundamental components of emotion. There is an increasing interest in the link between experiential and physiological processes across different disciplines, e.g., psychology, economics, or computer science. However, the findings largely rely on sample sizes that have been modest at best (limiting the statistical power) and capture only some concurrent biosignals. We present a novel publicly available dataset of psychophysiological responses to positive and negative emotions that offers some improvement over other databases. This database involves recordings of 1157 cases from healthy individuals (895 individuals participated in a single session and 122 individuals in several sessions), collected across seven studies, a continuous record of self-reported affect along with several biosignals (electrocardiogram, impedance cardiogram, electrodermal activity, hemodynamic measures, e.g., blood pressure, respiration trace, and skin temperature). We experimentally elicited a wide range of positive and negative emotions, including amusement, anger, disgust, excitement, fear, gratitude, sadness, tenderness, and threat. Psychophysiology of positive and negative emotions (POPANE) database is a large and comprehensive psychophysiological dataset on elicited emotions.

## Abstract (summary)

**Table:** 

Measurement(s)	affect • hemodynamic measures • electrodermal activity • fingertip skin temperature • Respiratory Pattern
Technology Type(s)	electronic rating scale • electrocardiography • impedance cardiography • hemodynamic measurement • electrodermal activity measurement • skin temperature sensor • Piezo-electric belt
Factor Type(s)	elicited emotion
Sample Characteristic - Organism	Homo sapiens
Sample Characteristic - Environment	laboratory environment

Machine-accessible metadata file describing the reported data: 10.6084/m9.figshare.17061512

## Background & Summary

The emotional response involves changes in subjective experience and physiology that mobilize individuals towards a behavioral response^1–6^. Theorists have debated for decades on the psychophysiology of human emotions focusing on several questions^7–10^. For instance, whether specific emotions produce a specific physiological response^3^, how different biosignals are correlated within an emotional response^5,6^, whether the physiological response allows predicting concurrent subjective experience^11^, what new features within a specific biosignal (e.g., the ECG wave) are influenced by emotions^12^, what improved methods of data processing can be used^13^, how emotions influence physiological patterns related to health^14,15^.

Physiological responses to the emotional stimuli were primarily of interest in psychology. However, emotions have recently also gained attention in other scientific fields, such as neuroscience^16^, product and experience design^17^, and computer science^18^. For instance, Affective Computing (an interdisciplinary field also known as Emotional AI) uses psychophysiological signals for developing algorithms that allow detecting, processing, and adapting to others’ emotions^19,20^. To allow machines to learn about specific emotion features, researchers have to provide these machines with multiple descriptors of emotional response, including subjective experience of affect (e.g., valence and motivational tendency) and objective physiological measures (e.g., cardiovascular, electrodermal, and respiratory measures).

These basic science and applied problems require robust empirical material that provides a large and comprehensive dataset that offers abundant emotions, diverse physiological signals, and the number of participants providing high statistical power. Moreover, researchers use various methods to elicit emotions^21^, including film clips^22,23^, pictures^24^, video recording/social pressure^25,26^, and behavioral manipulations^27^. Thus, accounting for various methods of emotion elicitation might contribute to database versatility.

A considerable amount of work has been done during the last two decades for creating multimodal datasets with psychophysiological responses to affective stimuli, including DEAP^28^, RECOLA^29^, CASE^30^, or K-EmoCon^31^. The strengths of our database – Psychophysiology of Positive and Negative Emotions (POPANE)^32^ are:

1. a wide range of positive and negative emotions, including amusement, anger, disgust, excitement, fear, gratitude, sadness, tenderness, and threat;
2. multiple methods to elicit emotions, namely: films, pictures, and affective social interactions (anticipated social exposition or expressing gratitude)
3. continuous emotional responses via self-reports and autonomic nervous system (ANS) activity using electrocardiography, impedance cardiography, electrodermal activity sensors, photoplethysmography (the hemodynamic measures), respiratory sensors, and a thermometer;
4. the length of our data is up to 725 hours of recordings, depending on the signal type. Table 1 presents the signal length for specific measures, stimuli, and emotion categories.

**Table 1:** Overview of Database Characteristics.

Emotion	Stimuli type	Stimuli Name	Used in Study	Time Interval [s]	*N*	Minutes of recordings of psychophysiological signals										
						Valence	Motivation	ECG	ICG	EDA	SBP	DBP	CO	TPR	RESP	TEMP
Amusement	film	A Fish called Wanda	5–7	120	348	270	426	656	526	270	212	212	212	212	0	0
	film	Benny & Joone	6	120	68	136	0	100	100	134	116	116	116	116	0	0
	film	The Visitors	5, 6	120	137	274	0	234	104	274	218	218	218	218	0	0
	film	When Harry Met Sally	5, 6	120	137	274	0	236	106	268	216	216	216	216	0	0
Anger	film	American History X	5–7	120	356	286	426	678	536	282	230	230	230	230	0	0
	film	In the Name of the Father	5, 6	120	137	274	0	242	100	270	218	218	218	218	0	0
	film	Man Bites Dog	5, 6	120	148	296	0	256	114	290	240	240	240	240	0	0
	speech preparation	Anger Speech	2	180	134	0	402	402	0	402	384	384	261	261	129	402
Disgust	film	Seven	6	120	73	146	0	106	106	142	126	126	126	126	0	0
Excitement	film	Summer Olympic Games	7	120	213	0	426	426	426	0	0	0	0	0	0	0
Fear	film	Fear Clips^A^	4	221	43	158	0	158	0	158	140	140	140	140	0	0
	film	The Blair Witch Project	6	120	68	136	0	106	106	130	118	118	118	118	0	0
Gratitude	sms	Gratitude Message	3	180	147	426	0	426	426	420	405	405	405	405	0	0
Neutral	film	Blue 1	5–7	120	324	222	426	624	498	222	190	190	190	190	0	0
	film	Blue 2	6	120	48	96	0	72	72	96	86	86	86	86	0	0
	film	Blue 3	6	120	48	96	0	72	72	96	86	86	86	86	0	0
	film	Emperor 1	5, 6	120	111	222	0	198	72	222	190	190	190	190	0	0
	film	Emperor 2	6	120	48	96	0	72	72	96	86	86	86	86	0	0
	film	The Lover	5	120	63	126	0	126	0	126	104	104	104	104	0	0
	film	Twin Peaks	6	120	48	96	0	72	72	96	86	86	86	86	0	0
	films	Neutral clips^B^	4	221	40	147	0	147	0	147	133	133	133	133	0	0
	pictures	Set of NAPS neutral photos	1, 2	180	60	0	180	180	0	180	168	168	168	168	0	180
	resting baseline	Physiological baseline	1–7	300^C^	1157	2771	2705	5236	2186	4390	3960	3960	3250	3250	710	1640
	SMS	Neutral message	3	180	147	426	0	426	426	420	405	405	405	405	0	0
Positive Emotion HA	pictures	Set of NAPS positive HA photos	1, 2	180	112	0	336	336	0	336	321	321	171	171	141	336
Positive Emotion LA	pictures	Set of NAPS positive LA photos	1, 2	180	113	0	339	339	0	339	333	333	189	189	138	339
Sadness	film	Dangerous Minds	6	120	61	122	0	94	94	116	106	106	106	106	0	0
	film	The Champ	7	120	213	0	426	426	426	0	0	0	0	0	0	0
Tenderness	film	Life Is Beautiful	6	120	69	138	0	106	106	136	118	118	118	118	0	0
	film	The Dead Poets Society	6	120	66	132	0	90	90	132	116	116	116	116	0	0
Threat	speech preparation	Threat Speech	1, 2	30^D^	95	0	285	285	0	285	267	267	267	267	0	285
				Total (hours)	123	106	215	114	175	156	156	138	138	19	53	123

Valence = positive-negative, Motivation = approach-avoidance tendency, Temp = fingertip skin temperature, Resp = respiration, ECG = electrocardiography, EDA = electrodermal activity, ICG = impedance cardiography, Z0 = baseline impedance, dZ = sensed impedance signal, dZ/dt = sensed impedance signal derivative over time, SBP = systolic blood pressure, DBP = diastolic blood pressure, CO = cardiac output, TPR = total peripheral resistance. HA = high-approach. LA = low-approach. ^A^Fear Clips: Blair Witch Project & A Tale of Two Sisters. ^B^Neutral clips: The Lover & Blue 2. ^C^in Study 3 baseline interval was 180s. ^D^in Study 2 threat speech preparation interval was 180s.

**Fig. 1:**
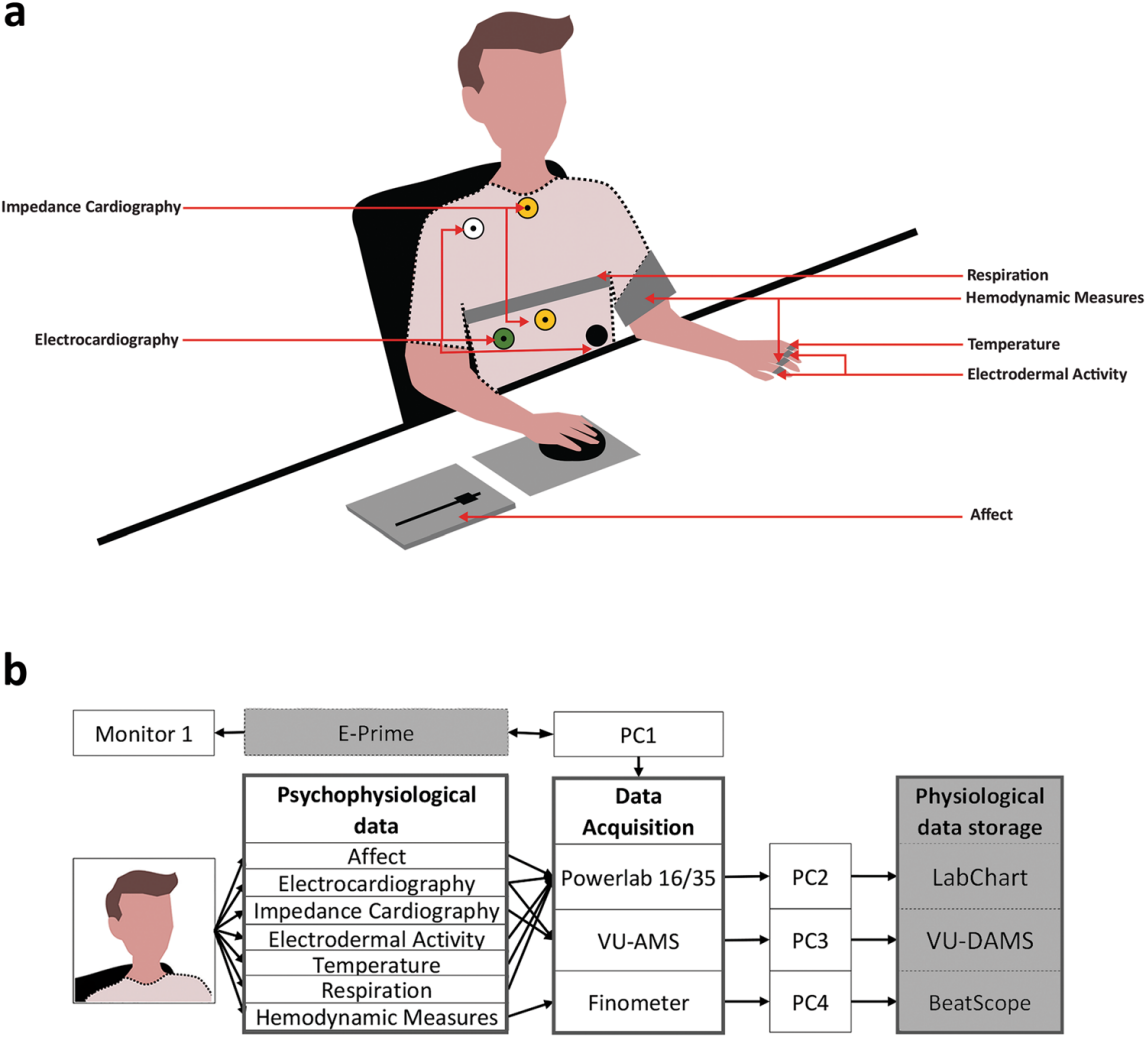
A schematic visualization of the data acquisition procedure. Panel a presents the approximate placement of the sensors. Panel b presents hardware (in white) and software (in grey) used for data acquisition. This figure was created by Katarzyna Janicka. The copyright of the figure is held by Katarzyna Janicka.

POPANE contains psychophysiological data from seven large-scale experiments that investigated the functions of positive and negative emotions. The studies tested how emotions influence the speed of cardiovascular recovery (Study 1 & 2)^33^, motivation to engage in a positive psychological intervention (Study 3)^34^, economic decisions (Study 4)^35^, responses to others successes (Study 5)^36^, responses to an unfair offer (Study 6)^37^, and gaming efficacy (Study 7)^38,39^. Tables 1 and 2 summarize the POPANE dataset. Figure 1 presents a schematic overview of the experimental setup used to collect the data.

**Table 2:** Overview of Studies Characteristics.

Study ID	Sample characteristics			Study characteristics			
	Size (% female)	Mean age (SD)	Participant pool	Study procedure	Method	Originally elicited emotions	Measures and signals
1	142 (75)	21.90 (2.49)	Undergraduates	Baseline, speech preparation, pictures presentation, recovery	Pictures and speech preparation	High-approach positive emotions, low-approach positive emotions, neutral conditions, threat	Motivation, ECG, EDA, Temp, Resp, SBP, DBP
2	186 (53)	21.96 (2.30)	Undergraduates	Baseline, pictures presentation, speech preparation, recovery	Pictures and speech preparation	High-approach positive emotions, low-approach positive emotions, neutral conditions, threat, anger	Motivation, ECG, EDA, Temp, SBP, DBP, CO, TPR
3	147 (50)	21.06 (1.91)	Undergraduates	Baseline, message writing 1, message writing 2	Interpersonal communication	Positive emotions (gratitude) and neutral conditions	Valence, ECG, EDA, ICG, SBP, DBP, CO, TPR
4	83 (59)	20.18 (1.88)	Undergraduates	Baseline, film clip, economic decision	Film clips	Negative emotions (fear) and neutral conditions	Valence, ECG, EDA, SBP, DBP, CO, TPR
5	199 (51)	22.38 (2.61)	Romantic couples	Baseline, three rounds of film clips, and responses to partner success (capitalization)	Film clips	Positive emotions (amusement), negative emotions (anger), and neutral conditions	Valence, ECG, EDA, SBP, DBP, CO, TPR
6	187 (53)	21.52 (2.73)	Undergraduates	Baseline, series of six film clips (all positive, negative, neutral, or mixed), ultimatum game	Film clips	Positive emotions (amusement, tenderness), negative emotions (anger, disgust, fear, sadness), and neutral conditions	Valence, ECG, EDA, ICG, SBP, DBP, CO, TPR
7	213 (0)	23.82 (3.57)	Gamers	Baseline, five rounds of film clips (random order), video-game match, recovery	Film clips	Positive emotions (amusement and excitement), negative emotions (anger and sadness), and neutral conditions	Motivation, ECG, ICG,

Valence = positive-negative, Motivation = approach-avoidance tendency, Temp = fingertip skin temperature, Resp = respiration, ECG = electrocardiography, EDA = electrodermal activity, ICG = impedance cardiography, Z0 = baseline impedance, dZ = sensed impedance signal, dZ/dt = sensed impedance signal derivative over time, SBP = systolic blood pressure, DBP = diastolic blood pressure, CO = cardiac output, TPR = total peripheral resistance.

## Methods

We present data collected in the Psychophysiological Laboratory, at the Faculty of Psychology and Cognitive Science, Adam Mickiewicz University, from November 2016 to July 2019 in Poznan, Poland. All methods are described in detail in the following works: Study 1 & 2^33^, Study 3^34^, Study 4^35^, Study 5^36^, Study 6^37^, Study 7^38^.

### Participants

The database includes 1157 cases (45% female) between the ages of 18 and 38 (*M* = 22.01, *SD* = 2.80). Table 2 presents participants’ characteristics for each study. We recruited participants via advertisements on Facebook and internal University communication channels. We asked participants to reschedule if they felt sick or experienced a serious negative life event and to abstain from vigorous exercise, food, and caffeine for two hours before testing. In recruitment, we invited participants that: 1) were healthy – had no significant health problems, 2) did not use drugs nor medications that might affect cardiovascular functions, 3) had no prior diagnosis of cardiovascular disease or hypertension. We introduced the above exclusion criteria to limit factors that might influence cardiovascular functions. We measured the participants’ height using an anthropometer and weight using an Omron BF511 scale (Omron Europe B.V., Netherlands). Each participant provided written informed consent and received vouchers for a cinema ticket for participation in the study. Of the participants, 895 participated in a single study, 101 in two studies, 19 in three studies, and two in four studies. The participants’ numerical IDs are presented in a metadata file. Next to specific within-study ID, we present the participants’ IDs from other studies so that within-person analyses might be possible to perform.

### Ethics statement

All studies were approved by and performed in accordance with guidelines and regulations of the Institutional Ethics Committee at the Faculty of Psychology and Cognitive Science, Adam Mickiewicz University.

### Procedure

#### Procedures common across the studies

In most of our studies, participants were tested individually in a sound-attenuated and air-conditioned room. Study 5 involved opposite-sex couples tested together in the same room but in separate cubicles, with no interaction with each other. Participants were randomly assigned to the experimental conditions. We also randomized the order of affective stimuli within the studies. Detailed information on the order of affective stimuli in each study is available in the metadata file. All instructions were presented, and responses were collected via a PC with a 23-inch screen. The experiments were run in the e-Prime 2.0 (Study 1, 2, 3, 4) and 3.0 (Study 5, 6, 7) Professional Edition environment (Psychology Software Tools).

Upon arrival in the lab, participants provided informed consent, and the researcher applied sensors to obtain psychophysiological measurements. Studies began with a five-minute resting baseline (only Study 3 began with a three-minute baseline). During baseline, participants were asked to sit and remain still. Upon completing all studies, biosensors were removed, and the participants were debriefed.

#### Study 1

After the baseline, participants completed the speech preparation task, which aimed at threat elicitation. Later, depending on randomization, affective pictures were presented on the PC screen to elicit high-approach motivation positive affect, low-approach motivation positive affect, or the neutral state for three minutes.

#### Study 2

After the baseline, participants watched affective pictures (high-approach positive affect, low-approach positive affect, or neutral depending on randomization) for three minutes. Afterward, they were asked to prepare the speech which aimed at threat or anger elicitation (depending on randomization).

#### Study 3

After completing the baseline, participants were requested to send two text messages: one expressing gratitude and one neutral. The order in which the messages were sent was counterbalanced. Before sending each SMS, participants were instructed to relax for three minutes and report their appraisals. Next, for another three minutes, participants were asked to think about a person to whom they were grateful for something. Afterward, participants were asked to send the message and wait for three minutes as the time needed for physiological recovery.

#### Study 4

Participants were told that they would be participating in two unrelated studies. The purpose of the first study was presented as determining the relationship between language orientation and the psychophysiological reactions to film clips. The purpose of the second study was presented as evaluating consumer products. After baseline, participants solved linguistic tasks. Next, they watched fear or neutral state eliciting film clips (depending on randomization). After the emotion manipulation, participants reported social needs and evaluated six pairs of commercial products.

#### Study 5

After completing the baseline, each participant was told to wait for their partner who would solve complex tasks. In fact, there were no tasks to be actually solved by any of the participants. Next, each participant completed three rounds consisting of 1) two minutes of watching the film clips while waiting for the partner; 2) receiving bogus information about the partners’ success; and 3) sending the feedback. Participants watched one of the three film sets, including only positive emotions, negative emotions, or a neutral condition (depending on randomization). The film clips were presented in a counterbalanced order.

#### Study 6

After the baseline, participants watched one of four 12-minute films’ presentations eliciting only positive emotions, only negative emotions, the mix of positive and negative emotions, and neutral states (depending on randomization). After watching the set of films, participants were instructed to play an ultimatum social game^40^. Participants received the offer, which was considered unfair by most people taking part in this type of research (“6 USD for me and 0.80 USD for you”). Next, participants were asked to decide to accept or reject the offer. Before receiving the offer and after deciding to accept or reject the offer, there was a 2-minute waiting period for recording physiological processes.

#### Study 7

After the baseline, participants completed five rounds consisting of (1) a 2-minute resting period; (2) 2-minute emotion elicitation (watching a film clip); (3) self-reports; and (4) playing a FIFA 19 match.

### Affective stimuli

#### Pictures

Study 1 and 2 used validated pictures^33^ from the Nencki Affective Pictures System^24^. We chose three sets of pictures to elicit: high-approach positive affect (Faces340; Landscapes008, L023, L100, L110, L117, L,140, L149, Objects078, O081, O096, O183, O254, O291, O323, People108, P173, P189), low-approach positive affect (Animals099, A153, Faces076, F113, F179, F228, F232, F234, F238, F330, F332, F337, F344, F347, F353, F358, Objects192, O260), and neutral experience (Faces157, F166, F167, F309, F312, Landscapes012, L016, L024, L056, L061, L067, L076, L079, Objects112, O204, O210, O310, O314). Study 1 & 2 used the same set of pictures.

#### Speech preparation

In Study 1, we elicited threat with a well-validated social threat protocol^25,26,41^. Participants were asked to prepare a 2-minute speech on the topic “Why are you a good friend?”. We informed participants that the speech would be recorded. Furthermore, participants received the information that they would be randomly selected to deliver the speech or not after the 30 s of speech preparation. However, after 30 s of preparations (anticipatory stress), each participant was informed that they were selected not to deliver the speech.

In Study 2, we randomly assigned participants to prepare a threat or anger-related speech. We used a similar method to elicit a threat as in Study 1, but participants were given 3 min to prepare the speech. Study 2 also intended to elicit anger with a similar method, i.e., anger recall task^42–44^. Participants were asked to prepare a speech on the topic “What makes you angry?”. Participants had 3 minutes to prepare the speech. After 3 minutes of both threat- and anger-related speeches, we informed participants that they were selected not to deliver the speech.

#### Interpersonal communication

In Study 3, participants expressed their gratitude (a positive relational emotion) towards their benefactors via texting. This intervention was developed within the field of positive psychology^45^. Participants express their gratitude towards their acquaintance by sending a text message during the laboratory session (Gratitude Texting). This intervention involved the essential elements of gratitude expression, including identification and appreciation of a good event, recognition of the benefactors’ role in generating the positive outcome, and the act of communicating gratitude itself^46^. In the control condition, we asked participants to send a neutral text message to their acquaintance with no suggestion regarding the topic. The control condition accounts for psychophysiological responses associated with texting in general^47^. Participants prepared their messages for three minutes.

#### Films

We used validated and reliable film clips selected from emotion-eliciting video clip databases^22,23,48–50^. Each clip lasted two minutes (except for films in Study 4 that, in sum, lasted for 3 minutes 41 seconds). Most of the film clips were short excerpts from commercially available films. Within the sessions, clips were presented in a counterbalanced order. Table 1, along with the metadata spreadsheet, presents which films were used to elicit emotions in the studies. The names of the film descriptions used for emotion elicitation are also available in the metadata file (the “*stimuli*” spreadsheet).

We elicited positive emotions with the following film clips: 1) *A Fish Called Wanda* (Surprisingly, the homeowners get inside and discover Archie dancing while naked); 2) *The Visitors* (Visitors damage the letter carrier’s car); 3) *When Harry Met Sally* (Sally pretends to have an orgasm in a restaurant); 4) *The Dead Poets Society* (Students climb on their desks to show their solidarity with their professor); 5) *Life Is Beautiful* (In a second world war prisoner’s camp, a father and a boy talk to the mother through a loudspeaker); 6) *Benny & Joone* (Benny plays dumb in the café); and 7) *Summer Olympic Games* (Athletes performing successfully and showing their joyful reactions). We used films 1–3 in Study 5, films 1–6 in Study 6, and films 1 & 7 in Study 7.

We elicited negative emotions with the following film clips: (1) *The Blair Witch Project* (the characters die in an abandoned house); (2) *A Tale of Two Sister*s (the clip begins with suspense and ends with an intense explosion) (3) *American History X* (A neo-nazi kills Blackman’s by smashing his head on the curb); (4) *Man Bites Dog (*A hitman pulls out a gun, yelling at an elderly woman); (5) *In the Name of the Fathe*r (Interrogation scene); (6) *Seven* (the police find a decomposing corpse); (7) *Dangerous Minds* (The teacher informs the class about the death of their classmate); and (8) *The Champ* (the boy cries after his father dies). We used films 1 & 2 in Study 4, films 1 & 3–7 in Study 6, films 3–5 in Study 5, and films 3 & 8 in Study 7.

For neutral conditions, we used the following film clips: (1) *Blue* 1 (A man organizes the drawers in his desk, or a woman walks down an alley); (2) *The Lover* (The character walks around town); (3) *Blue* 3 (The character passes a piece of aluminum foil through a car window); (4) *The Last Emperor* 1 (Conversation between the Emperor and his teacher); (5) *Blue* 2 (A woman rides up on an escalator, carrying a box); (6) *The Last Emperor* 2 (City life scenes); (7) *Twin Peaks: Fire Walk with Me* (the character sweeps the floor in the bar). We used films 2 & 5 in Study 4, films 1, 3 & 4 in Study 5, films 1 & 3–7 in Study 6, and film 5 in Study 7.

### Sensors & instruments

We present sensors and instruments used in our studies with examples illustrating their possible research applications.

#### Affect

Participants reported the affective experience to the emotional stimuli continuously with an electronic rating scale^51^. We investigated two dimensions of affect: valence (Study 3, 5, & 6) and approach/avoidance motivational tendency (Study 1,2, & 7). Valence is the degree of feeling pleasure or displeasure in response to a stimulus (e.g., object, event, or a person). Individuals experience positive valence while facing favorable objects or situations (e.g., smiling people or amusing events), and negative valence while facing unfavorable objects or situations (e.g., sad individuals)^24^. The approach/avoidance motivational tendency is the urge to move toward or away from an object^52^. Individuals experience high-approach motivation while facing desirable or appetitive objects or situations (e.g., delicious food or sexually attractive individuals), and high-avoidance motivation while facing undesirable or aversive objects or situations (e.g., accidents or infected individuals). We focused on valence because it is the most fundamental and well-studied dimension of the affect, and we focused on the approach/avoidance motivational tendency that is a rather novel dimension considered in the literature that might advance understanding emotions’ functions^53^.

Participants reported valence on a scale from 1 (*extremely negative*) to 10 (*extremely positive*) or approach/avoidance motivational tendency on a scale from 1 (*extreme avoidance motivational tendency*) to 10 (*extreme approach motivational tendency*). Participants were asked to adjust the rating scale position as often as necessary so that it always reflected how they felt at a given moment. For valence, we asked the participants to move the tag to the right side of the scale when they felt more positive or pleasant and to move the tag to the left side of the scale when they felt more negative or unpleasant. For the approach/avoidance motivational tendency, we asked the participants to move the tag to the right side of the scale when they felt the motivation to go toward or engage with the stimulus and to move the tag to the left side of the scale when they felt the motivation to go away or disengage with the stimulus. Previous research indicated that rating scales are valid for reporting the intensity of valence and approach/avoidance motivation^24,51,54^.

The signal was sampled at a rate of 1 kHz by Powerlab 16/35 (ADInstruments). Furthermore, we provided a validated positive-negative (Study 3–6) or approach-avoidance (Study 1,2 & 7) graphical scale modeled after the self-assessment manikin above the numeric scale^38,55^.

#### Electrocardiography

We used two electrocardiographs (ECG), BioAmp with Powerlab 16/35 AD converter (ADInstruments, New Zealand) (Study 1,2,4 & 5) and Vrije Universiteit Ambulatory Monitoring System (VU-AMS, the Netherlands) (Study 3, 6 & 7). We used pre-gelled AgCl electrodes placed in a modified Lead II configuration. The signal was stored on a computer with other biosignals using a computer‐based data acquisition and analysis system (LabChart 8.1; ADInstruments or VU-AMS Data, Analysis & Management Software; VU-DAMS 3.0). The ECG signal was sampled at a frequency of 1 kHz. ECG signal allows the computation of numerous indexes with the most popular involving 1) heart rate, which reflects the autonomic arousal, associated with, e.g., dually innervated sympathetic nervous system (SNS) and parasympathetic nervous system (PNS) activity, and is related to motivational intensity, action readiness, and engagement^56,57^, and 2) heart rate variability linked with stress, self-regulatory efforts, and recovery from stress^58^.

#### Impedance Cardiography

We recorded the impedance cardiography (ICG) signal continuously and noninvasively with the Vrije Universiteit Ambulatory Monitoring System (VU-AMS, the Netherlands) following psychophysiological guidelines^59,60^. We used pre-gelled AgCl electrodes placed in a four-spot electrode array for ICG^59^. The signal was stored on a computer with other biosignals using a computer‐based data acquisition and analysis system (VU-DAMS 3.0). The ICG signal was sampled at a frequency of 1 kHz. ICG provided three channels: baseline impedance (Z0), sensed impedance signal (dZ), and its derivative over time (dZ/dt). In addition to ECG signal, ICG signal allows the computation of indexes linked to the pace and blood volume of the heartbeats, including (1) pre-ejection period reflecting sympathetic cardiac efferent activity which is associated, e.g., with motivational intensity and engagement^56,57^; (2) stroke volume which is linked with stress^61^; and (3) cardiac output which is used, e.g., to discriminate between challenge vs. threat stress response^62^.

#### Hemodynamic measures

We recorded hemodynamic responses using two models of the Finometer: Finometer MIDI (Finapres Medical Systems, Netherlands) (Study 1, 2, 4 & 5) and Finometer NOVA (Finapres Medical Systems, Netherlands) (Study 3, 5 & 6). Finometers provided systolic blood pressure (SBP), diastolic blood pressure (DBP), cardiac output (CO), and total peripheral resistance (TPR). SBP, DBP, CO, TPR were recorded continuously beat-by-beat (only in Study 1, we recorded SBP and DBP as a raw signal). Finometers use the volume-clamp method first developed by Penaz^63^ to measure finger arterial pressure waveforms with finger cuffs. The data were exported to the Powerlab 16/35 data acquisition system (ADInstruments, New Zealand) and LabChart 8.1 (ADInstruments, New Zealand) (Study 1, 3 & 4) or collected with BeatScope 2.0 (Finapres Medical Systems, Netherlands)^64^ (Study 2, 5 & 6). SBP and DBP is used to assess, e.g., effort investment^54^ or cardiovascular health risk^65^, whereas CO and TPR are used to differentiate between challenge vs. threat stress response^56^.

#### Electrodermal activity

We recorded the electrodermal activity (EDA) with the GSR Amp (ADInstruments) at 1 kHz. We used electrodes with adhesive collars and sticky tape attached to the medial phalanges of digits II and IV of the left hand. The electrodes had a contact area of 8 mm diameter and were filled with a TD‐246 sodium chloride skin conductance paste. The signal was stored on a computer with other biosignals using a computer‐based data acquisition and analysis system (LabChart 8.1; ADInstruments). Skin conductance reflects beta-adrenergic sympathetic activity, and some examples of its use comprise mental stress, cognitive load, and autonomic arousal^66^.

#### Respiration

In Study 1, we recorded respiratory action with a piezo-electric belt, Pneumotrace II (UFI, USA), sampled at 1 kHz. The belt was attached around the upper chest near the level of maximum amplitude for thoracic respiration. The signal was stored on a computer with other biosignals using a computer‐based data acquisition and analysis system (LabChart 8.1; ADInstruments). The respiratory action allows the computation of respiratory rate and depth associated, e.g., with mental stress^67^, arousal^68^, and increases in negative emotion, e.g., anger and fear^5^.

#### Fingertip skin temperature

In Study 1, we measured fingertip temperature with a temperature probe attached to a Thermistor Pod (ADInstruments, New Zealand). The thermometer was attached at the distal phalange of the left hand’s V finger, sampled at 1 kHz. The signal was stored on a computer with other biosignals using a computer‐based data acquisition and analysis system (LabChart 8.1; ADInstruments). Changes in digit temperature reflect sympathetically innervated peripheral vasoconstriction and vasodilation that decreases or increases the fingertip temperature due to lower or higher blood supply. For instance, the fingertip temperature decreases in response to stress^69^ and increases in response to joy^70^. Fingertip temperature is usually lower than other body temperature measures, e.g., the axillary or oral temperature^71^. Moreover, fingertip skin temperature can be much lower for some participants due to individual differences in hand morphology as well as ambient temperature. For instance, thermoregulatory cold-induced vasodilation occurs when hands are exposed to cold weather in winter^72^.

### Data acquisition

Figure 1 presents the experiments and the data acquisition setup. Stimuli were managed through E-Prime (Psychology Software Tools, Inc.). E-Prime software sent the markers to the data acquisition devices (LabChart and VU-AMS), by which we were able to synchronize and merge the recordings from different devices into a single data file. The rating scale, ECG, EDA, thermometer, and respiratory belt were directly connected to the Powerlab 16/35 and then to the acquisition personal computer (PC) over a USB port. The ECG and ICG were directly connected to the VU-AMS and then to the acquisition PC3 over a USB port. The blood pressure measures were collected via a finger cuff directly connected to the Finometers and then to the acquisition PC over a USB port. We synchronized LabChart and VU-AMS with Finometer data by manually adding the markers at the same time during data recording. Data were managed in the following manner: 1) Powerlab data was stored in LabChart 8.0; 2) VU-AMS data was stored in VU-DAMS; and 3) Finometer data were stored in BeatScope. The acquired data from each participant was exported with the timestamp provided by the acquisition PC and markers into the TXT data files.

### Data preprocessing

Physiological data collected across seven studies were exported from the acquisition formats by the first author [MBe]. The participants’ number differs from the initial studies due to various issues such as device malfunction, signal artifacts, and missing data files. We presented data that had high signal quality. Thus, some participants’ data from some channels (devices) were excluded, resulting in an 8% decrease in the participants’ pool.

**Fig. 2:**
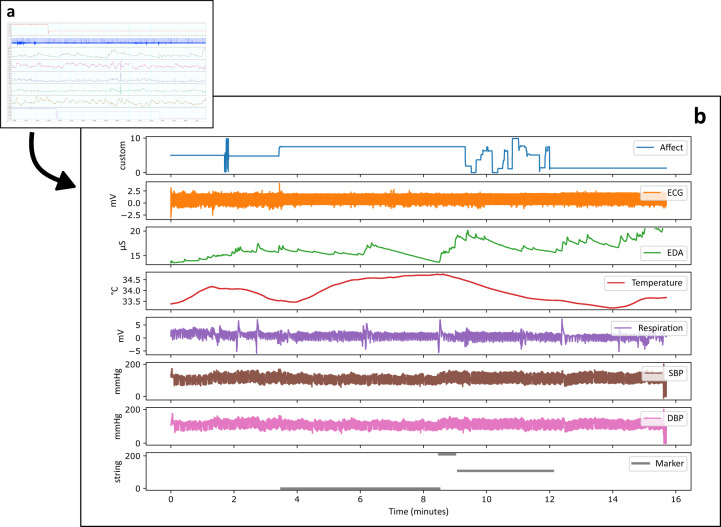
Schematic presentation of data preprocessing. The data were first exported from the acquisition software (panel a) and then preprocessed and integrated into CSV files. The resulting CSV files can be easily loaded into most statistical software and packages, such as IBM’s SPSS or Python Pandas & SciPy modules (for visualization in Python’s Pandas module, see panel b).

The exported TXT, CSV, and metadata files were preprocessed using Python^73,74^ scientific libraries (e.g., pandas 1.1.5, numpy 1.19.2; see Code Availability, for detailed information) (Fig. 2a). All signals were resampled to 1 kHz, using the previous neighbor interpolation method (Fig. 2b). Signals from different devices were time-synchronized using synchronization markers generated during experiments. We marked the baselines and emotion elicitations within the files. Finally, data across studies were exported to a normalized form, consisting of a header, a predefined file structure, and a standardized subject naming convention.

## Data Records

The POPANE dataset is publicly available at the *Open Science Framework* repository^32^.

### Metadata

We present auxiliary information about the experiments in the metadata spreadsheet. The metadata file includes participants’ ID, sex, age, height, weight, experimental conditions for each study, stimuli order within a session, and information about missing data, outliers, and artifacts (sheets “Study 1–7”). Furthermore, the metadata file provides information on individuals that participated more than once across the studies by showing all their study-related IDs. The description of labels used for tagging discrete emotions is also available in the metadata file (the “*stimuli*” spreadsheet).

### Dataset structure

The data repository consists of seven ZIP-compressed directories (folders), one for each study, e.g., “Study1” directory was compressed to “Study1.zip” archive file, “Study2” was compressed as “Study2.zip”, etc. Each of these directories contains a set of CSV files with psychophysiological information for particular subjects. We used a consistent CSV file naming convention, i.e.: “S < study_id > _P < participant_id > _ < phase_name > .csv”, where “S” stands for study, “P” for participants, e.g.: S1_P10_Baseline.csv, or S6_P4_Amusement1.csv. The “ < study_id > ” & “ < particpant_id > ” are natural numbers identifying a study and a participant. The “ < phase_name > “ is the name of the phase of an experiment, e.g., “Baseline”, or “Amusement1”. The description of all experimental-phase labels is explained in the metadata spreadsheet. All psychophysiological signals recorded during the experiment for each individual are also available in a single CSV datafile named “S < study_id > _P < participant_id > _All.csv”. All the other files for a particular participant named in the following manner: “S < study_id > _P < participant_id > _ < phase_name > .csv” are files containing a subset of records (an excerpt) extracted from a basic “S < study_id > _P < participant_id > _All.csv” file. Thus, “S < study_id > _P < participant_id > _All.csv” files store either signals related to a particular experimental phase or signals gathered during time intervals, where no experimental conditions were present, i.e., signals that were not related to the affective manipulation.

Furthermore, we also included one additional component, i.e., “POPANE dataset”. This component contains a set of ZIP-compressed directories with a set of CSV files with psychophysiological information for particular participants, baselines, and emotions. We grouped the datafiles from all studies into a single folder sorted by emotions. This simplifies the usage of our dataset as the single set of emotion-related data from all 1157 cases.

A sample from Study 1 is available for preview and testing and can be obtained from the data repository as “Study1_sample.zip”. The compressed sample file size is 42 MB (208 MB uncompressed), as compared to 2.0 GB (9.3 uncompressed) of the complete dataset for Study 1. This provides potential users of the dataset with an opportunity to get the notion of the data without downloading the whole dataset. For the visualization of these sample data, see Fig. 2b.

### Single file structure

Each of the CSV files in the dataset has a 11-line header, i.e., each file’s first eleven rows start with a hash sign (“#“). In the header, file metadata is available, including:

1. ID of the study as a variable “Study_name”, e.g., “Study_7”;
2. participant’s ID within the study as a variable “Subject_ID”, e.g., “119”;
3. participant’s age as a variable “Participant_Age”, e.g., “23”;
4. participant’s sex coded as man = 0, woman = 1, as a variable “Subject_Sex”;
5. participant’s height in centimeters as a variable “Participants_Height”, e.g. “178”;
6. participant’s weight in kilograms as a variable “Participants_Weight”, e.g. “74”;
7. channel/sensor name as a variable “Channel_Name”, e.g., “timestamp”, “affect”, “ECG”, “dzdt”, “dz”, “z0”, or “marker”;
8. category of the data in each column as a variable “Data_Category”, e.g., “timestamp”, “data”, and “marker”;
9. units of the measurement as a variable “Data_Unit”, e.g.: “second”, “millivolt”, or “ohm”;
10. sample rate of data collection as a variable “Data_Sample_rate”, e.g.: “1000 Hz”, or “beat to beat”;
11. name of the device (manufacturer) used for data collection as a variable “Data_Device”, e.g., “LabChart_8.19_(ADInstruments,_New Zealand)”, “Response_Meter_(ADInstruments,_New Zealand)”, or “ECG (Vrije_Universiteit_Ambulatory_Monitoring_System,_VU-AMS,_the Netherlands)”.

If no data are available for the participant’s age, sex, height, and weight, we inserted a value of “−1”.

Following the header, each CSV file contains 7–12 columns, depending on the study. For studies in which data were gathered from more channels, there are more columns in CSV files. Sensor names used in all studies are consistent across all CSV files (see the metadata file). The first column of the data table (except for the header) contains timestamps, as provided by a clock on the main data acquisition (logging) computer – the timestamp format is time in seconds. In the last column, there is a marker that identifies the specific phase of the experiment. The metadata file provides a full explanation of the stimulus IDs used to mark the specific phase of the experiment, e.g., “−1” indicates the experimental baseline, while “107” indicates the neutral film clip “The Lover”. The columns in between the timestamp and the marker contain the physiological data (see Table 3 for details).

**Table 3:** Datafile information and structure.

Study ID	File count (per parti-cipant	Types of < phase_name > (time interval in seconds)	Types of < EmotionName > in each study	Column Names	Number of columns
1	568 (4)	'All’, ‘Baseline’ (300), ‘Threat’(30), ‘ < EmotionName > ’(180)	'Neutral8’, ‘Positive_Emotion_High_Approach’, ‘Positive_Emotion_Low_Approach’, ‘Threat'	Timestamp, Affect, ECG, EDA, Temp, Respiration, SBP, DBP, marker	9
2	744 (4)	'All’, ‘Baseline’(300), ‘ < EmotionName1–2 > ’(180)	'Anger4’, ‘Neutral8’, ‘Positive_Emotion_High_Approach’, ‘Positive_Emotion_Low_Approach’, ‘Threat'	Timestamp, Affect, ECG, EDA, Temp, SBP, DBP, CO, TPR, marker	10
3	588 (4)	'All’, ‘Baseline’(180), ‘ < EmotionName1–2 > ’(180)	‘Gratitude’, ‘Neutral9’	Timestamp, Affect, ECG, EDA, DZ, DZ/DT, Z0, SBP, DBP, CO, TPR, marker	12
4	249 (3)	'All’, ‘Baseline’(300), ‘ < EmotionName > ’(221),	‘Fear2’, ‘Neutral10’	Timestamp, Affect, ECG, EDA, SBP, DBP, CO, TPR, marker	9
5	995 (5)	'All’, ‘Baseline’(300), ‘ < EmotionName1–3 > ’(120)	‘Amusement2’, ‘Amusement3’, ‘Amusement4’, ‘Anger1’, ‘Anger2’, ‘Anger3’, ‘Neutral1’, ‘Neutral6’, ‘Neutral7'	Timestamp, Affect, ECG, EDA, SBP, DBP, CO, TPR, marker	9
6	1496 (8)	'All’, ‘Baseline’(300), ‘ < EmotionName1–6 > ’(120)	‘Amusement1’, ‘Amusement2’, ‘Amusement3’, ‘Amusement4’, ‘Anger1’, ‘Anger2’, ‘Anger3’, ‘Disgust’, ‘Fear1’, ‘Neutral1’, ‘Neutral2’, ‘Neutral3’, ‘Neutral4’, ‘Neutral5’, ‘Neutral6’, ‘Sadness1’, ‘Tenderness1’, ‘Tenderness2'	Timestamp, Affect, ECG, EDA, DZ, DZ/DT, Z0, SBP, DBP, CO, TPR, marker	12
7	1491 (7)	'All’, ‘Baseline’(300), ‘ < EmotionName1–5 > ’(120)	‘Amusement4’, ‘Anger3’, ‘Excitement’, ‘Neutral6’, ‘Sadness2'	Timestamp, Affect, ECG, DZ, DZ/DT, Z0, marker	7

Affect = response meter measuring valence or motivation, Temp = fingertip skin temperature, ECG = electrocardiography, EDA = electrodermal activity, Z0 = baseline impedance, dZ = sensed impedance signal, dZ/dt = sensed impedance signal derivative over time, SBP = Systolic blood pressure, DBP = diastolic blood pressure, CO = cardiac output, TPR = total peripheral resistance, Number of columns = number of columns in the single .csv file after the header.

### Scripts

We used different acquisition programs; therefore, the exported data had to be integrated into a common format. An automatic preprocessing procedure was implemented in Python scripts. We converted the raw acquired data (obtained with a proprietary acquisition software) into a consistent format and saved it in CSV files. Consequently, data from several sources were integrated to be easily imported into all common statistical software packages. We also prepared examples in IPython Jupyter Notebooks presenting how to load and visualize psychophysiological data from sample files for Study 1. Both the conversion scripts and the Notebooks can be obtained from our source code repository available at GitHub: https://github.com/psychosensing/popane-2021.

## Technical Validation

### Qualitative validation

**Fig. 3:**
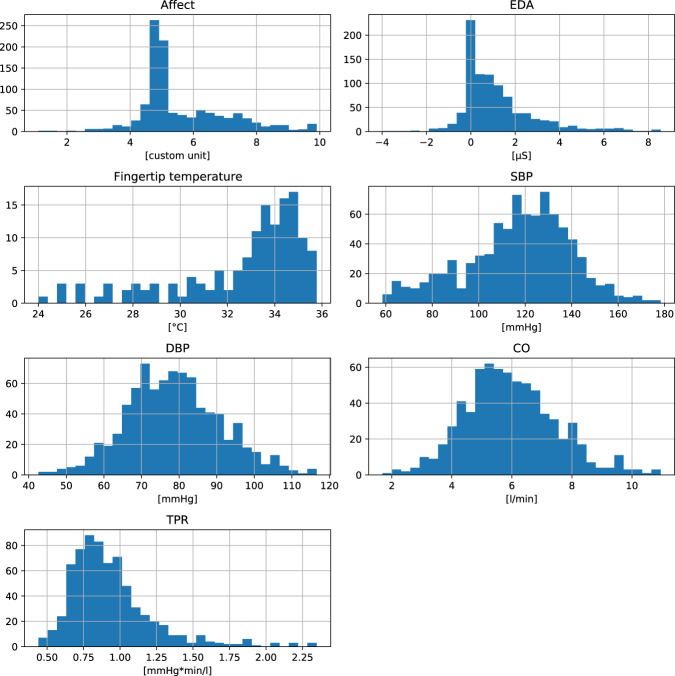
Data histograms of baseline psychophysiological levels. This figure presents the distribution of the mean psychophysiological levels for resting baseline but does not present raw recordings (e.g., ECG in mV) that require further processing (e.g., analysis to calculate HR or HRV).

The data quality was assured by following recommendations in affective science^3^. First, we used validated methods (e.g., protocols and stimuli) to elicit emotions in our experiments. We used stimuli in line with well-established methods in the affective science^21^. Second, the data were collected by experimenters that completed 30 h training in psychophysiological research provided by MBe and LDK. Third, prior to performing preprocessing, the first author (MBe) visually inspected all physiological signals. Before inclusion in the database, MBe manually double-checked all datasets for missing or corrupted data. Table 4 presents missing data for each stimulus and physiological signal. The histograms in Figure 3 show the distributions of the selected physiological signals during the resting baseline. Figure 3 also presents that collected signals had standard ranges. For instance, most participants presented a healthy SBP and DBP range during the resting baseline of the experiments^75^. This figure does not present raw recordings (e.g., ECG in mV) that require further processing (e.g., breathing rate based on peak analysis).

**Table 4:** Number of missing data.

Emotion	Stimuli type	Stimuli Name	Used in Study	Time Interval [s]	*N*	Number of missing data for psychophysiological signals										
						Valence	Motivation	ECG	ICG	EDA	SBP	DBP	CO	TPR	RESP	TEMP
Amusement	film	A fish called Wanda	5–7	120	348	0	0	20	20	0	29	29	29	29	—	—
	film	Benny & Joone	6	120	68	0	—	18	18	1	10	10	10	10	—	—
	film	The visitors	5, 6	120	137	0	—	20	20	0	28	28	28	28	—	—
	film	When Harry met Sally	5, 6	120	137	0	—	19	19	3	29	29	29	29	—	—
Anger	film	American History X	5–7	120	356	0	0	17	17	2	28	28	28	28	—	—
	film	In the name of the father	5, 6	120	137	0	—	16	16	2	28	28	28	28	—	—
	film	Man bites dog	5, 6	120	148	0	—	20	20	3	28	28	28	28	—	—
	speech preparation	Anger Speech	2	180	134	0	0	0	0	0	6	6	4	4	0	0
Disgust	film	Seven	6	120	73	0	—	20	20	2	10	10	10	10	—	—
Excitement	film	Summer Olympic Games	7	120	213	—	0	0	0	—	—	—	—	—	—	—
Fear	film	Fear Clips^A^	4	221	43	0	—	0	—	0	5	5	5	5	—	—
	film	The Blair Witch Project	6	120	68	0	—	15	15	3	9	9	9	9	—	—
Gratitude	sms	Gratitude Message	3	180	147	5	—	5	5	7	12	12	12	12	—	—
Neutral	film	Blue 1	5–7	120	324	0	0	12	12	0	16	16	16	16	—	—
	film	Blue 2	6	120	48	0	—	12	12	0	5	5	5	5	—	—
	film	Blue 3	6	120	48	0	—	12	12	0	5	5	5	5	—	—
	film	Emperor 1	5, 6	120	111	0	—	12	12	0	16	16	16	16	—	—
	film	Emperor 2	6	120	48	0	—	12	12	0	5	5	5	5	—	—
	film	The lover	5	120	63	0	—	0	0	0	10	10	10	10	—	—
	film	Twin Peaks	6	120	48	0	—	12	12	0	5	5	5	5	—	—
	films	Neutral clips^B^	4	221	40	0	—	0	—	0	4	4	4	4	—	—
	pictures	Set of NAPS Neutral photos	1, 2	180	60	—	0	0	—	0	4	4	4	4	—	0
	resting baseline	Physiological baseline	1–7	300^A^	1157	5	0	53	53	10	98	98	98	98	0	0
	sms	Neutral message	3	180	147	5	—	5	5	7	12	12	12	12	—	—
Positive Emotion HA	pictures	Set of NAPS Positive HA photos	1, 2	180	112	—	0	0	—	0	5	5	4	4	4	0
Positive Emotion LA	pictures	Set of NAPS Positive LA photos	1, 2	180	113	—	0	0	—	0	2	2	2	2	2	0
Sadness	film	Dangerous minds	6	120	61	0	—	14	14	3	8	8	8	8	—	—
	film	The champ	7	120	213	—	0	0	0	—	—	—	—	—	—	—
Tenderness	film	Life is beautiful	6	120	69	0	—	16	16	1	10	10	10	10	—	—
	film	The Dead Poets society	6	120	66	0	—	21	21	0	8	8	8	8	—	—
Threat	speech preparation	Threat Speech	1, 2	30^B^	95	—	0	0	—	0	6	6	6	6	—	0

Valence = positive-negative, Motivation = approach-avoidance tendency, Temp = fingertip skin temperature, Resp = respiration, ECG = electrocardiography, EDA = electrodermal activity, ICG = impedance cardiography, Z0 = baseline impedance, dZ = sensed impedance signal, dZ/dt = sensed impedance signal derivative over time, SBP = systolic blood pressure, DBP = diastolic blood pressure, CO = cardiac output, TPR = total peripheral resistance. HA = high-approach. LA = low-approach. ^A^Fear Clips: Blair Witch Project & A Tale of Two Sisters. ^B^Neutral clips: The Lover & Blue 2. ^C^in Study 3 baseline interval was 180s. ^D^in Study 2 threat speech preparation interval was 180s.

### Quantitative validation

We evaluated the quality of the signal with the Signal-to-Noise Ratio (SNR). In order to calculate SNR across the diverse physiological signals, we used an algorithm based on the autocorrelation function of the signal, using the second-order polynomial for fitting the autocorrelation function curve^76^. The script we used for calculating SNR is available in the project’s GitHub repository (https://github.com/psychosensing/popane-2021). We calculated SNR for all baselines and emotion elicitations across seven studies (Table 5). The calculated SNR indicated the high quality of all collected signals^77^, SNR_min_ = 5.67 dB, with mean SNR ranging from 37.82 dB to 67.39 dB depending on physiological signal and study. We identified outliers above SNRs’ *z*-scores higher than 3.29^78^, resulting in 290 parts (1.09% of all calculated SNR values) identified as SNR outliers. Next, the first author (MBe) visually inspected all flagged data to determine whether it should be classified as artifacts, resulting in 257 SNR outlying data points being identified as artifacts (88% of the low SNR data; less than 0.96% of all calculated SNR values). Both outliers and artifacts are presented in the metadata file.

**Table 5:** Means and Standard Deviations (in parentheses) for Signal-to-Noise Ratio (SNR) in decibels (dB) for particular signals acquired in 7 studies.

Study ID	Signal to Noise Ratio									
	ECG	DZ	Z0	EDA	SBP	DBP	CO	TPR	RESP	TEMP
1	22.11	—	—	51.62	43.50	44.02	—	—	47.25	52.45
	(8.56)			(4.26)	(1.28)	(1.13)			(5.07)	(4.28)
2	21.80	—	—	54.72	54.29	54.36	52.92	48.49	—	—
	(8.69)			(2.57)	(0.93)	(2.09)	(1.49)	(2.31)		
3	44.87	49.62	54.28	54.25	54.19	54.04	54.4	54.15	—	—
	(1.71)	(3.56)	(0.45)	(4.53)	(0.39)	(0.51)	(0.55)	(0.58)		
4	35.35	—	—	55.16	55.27	54.99	53.55	53.43	—	—
	(3.81)			(2.20)	(0.60)	(0.67)	(1.13)	(2.6)		
5	32.99	—	—	53.53	53.10	52.83	51.17	51.84	—	—
	(5.43)			(2.52)	(1.60)	(1.64)	(2.3)	(2.73)		
6	44.81	47.62	53.02	52.95	52.89	52.69	51.28	52.55	—	—
	(2.04)	(3.57)	(1.66)	(2.43)	(1.26)	(1.26)	(1.71)	(1.32)		
7	45.34	47.52	53.14	—	—	—	—	—	—	—
	(2.09)	(3.78)	(1.61)							

### Previous studies

For each study represented in the dataset, we ran manipulation checks that contributed to the technical validation. We found that the stimuli produced expected affective and physiological responses in participants^33–38^. For instance, in Study 5, we found that individuals who watched the positive film clips reported more positive valence, whereas individuals who watched the negative film clips reported more negative valence, compared to individuals who watched the neutral film clips^36^. Furthermore, individuals in the positive and negative emotion conditions displayed greater physiological reactivity (e.g., SBP and DBP) than individuals in the neutral conditions^36^.

## Usage Notes

The POPANE dataset is available at 10.17605/OSF.IO/94BPX. The data in the datasets are saved in CSV format. The dataset can be used to test hypotheses on positive and negative emotions, create psychophysiological models and/or standards, or as an example data for testing technical aspects of the analyses and/or validation of mathematical models. These data can be of interest for several scientific fields such as psychology, e.g., for investigating human emotions based on physiological and psychometric information, or computer science (machine learning) for implementing automatic emotion recognition, or clustering data related to particular emotions.

### Limitations

There are some shortcomings of our dataset. First, some data are missing because recordings for some of the participants could not be reliably collected due to technical reasons. Second, this dataset cannot be employed to investigate psychophysiological differences between ethnicities, neither between the group ages, as more than 99% of the participants were Caucasian young adults. This is an important limitation because some studies indicated physiological differences in baseline levels and reactivity to some stressors depending on the participant’s age^79,80^ and ethnicity^81^. Moreover, some studies in the dataset recruited only male participants. This is important to control if the whole dataset would be used for testing hypotheses regarding sex differences^82^. Third, our dataset does not include participants diagnosed with cardiovascular disease. However, we did not collect information about other health issues, e.g., psychiatric or neurological diagnosis.

Fourth, this dataset is *a posteriori* use of the previously acquired data in already published independent studies. However, some participants (12%) took part in more than one study. We identified these participants in the metadata file. Thus, if the whole dataset is used to test hypotheses, researchers should consider this issue. In contrast, some authors might be particularly interested in the use of repeated data collected from the same participants, e.g., to test intraperson stability or change.

Fifth, most of the film clips were short excerpts from commercially available films. Thus, some of our participants might have already been familiar with them.

Sixth, in our studies, we measured autonomic nervous system (ANS) reactivity to nine discrete emotions. This is not an exhaustive list of affective states related to ANS activity. Future studies may focus on emotions that are examined less often in psychophysiological studies, including pride, craving, love, or embarrassment^6^. Furthermore, the emotions elicited in our studies were not balanced in valence, as some studies were focused on the differences between neutral conditions and positive emotions (Study 3) or negative emotions (Study 4).

In summary, the POPANE database is a large and comprehensive psychophysiological dataset on emotions. We hope that POPANE will provide individuals, companies, and laboratories with the data they need to perform their analyses to advance the fields of affective science, physiology, and psychophysiology. We invite you to visit the project website https://data.psychosensing.psnc.pl/popane/index.hml.

### GitHub repository

Scripts for converting data from proprietary acquisition software formats into consistent CSV files, as well as IPython Jupyter Notebooks presenting how to load the data from POPANE CSV files into Python Pandas DataFrame structure are available at the following GitHub repository: https://github.com/psychosensing/popane-2021.

## Data Availability

The code can be accessed on the public GitHub repository: https://github.com/psychosensing/popane-2021. It is licensed under MIT OpenSource license, i.e., the permission is granted, free of charge, to obtaining a copy of this software and associated files (e.g., the Jupyter IPython Notebooks), subject to the following conditions: the copyright notice and the MIT license permission notice shall be included in all copies or substantial portions of the software based on the scripts we published. Scripts that we used to transform the data from proprietary acquisition formats into coherent CSV files utilized Python 3.6^83^. The list of the specific modules and their versions is available in the “requirements.txt” file in the GitHub repository. Jupyter Notebooks use Python version: 3.5.3, as well as the following Python modules: packages related to Jupyter Notebook: notebook module v. 6.1.4; jupyter-core module v. 4.6.3, jupyter-client v. 6.1.7; ipython v. 7.9.0; ipykernel v. 5.3.4^84^; and a data organization and manipulation module – pandas v. 0.25.3^73^.
